# Non-aromatizable androgens modulate the lipopolysaccharide induced expression of the P2X7 receptor in human adipocytes

**DOI:** 10.3389/fphar.2023.1251035

**Published:** 2023-10-23

**Authors:** Angelo Di Vincenzo, Marnie Granzotto, Marika Crescenzi, Roberto Vettor, Marco Rossato

**Affiliations:** Internal Medicine, Department of Medicine—DIMED, University Hospital of Padova, Padova, Italy

**Keywords:** human adipocytes, testosterone, estradiol, anastrozole, dihydrotestosterone, aromatase, lipopolysaccharide, P2X7 receptor

## Abstract

**Introduction:** The activation of the P2X7 receptor subtype (P2X7R) has a main role in orchestrating the cellular inflammatory response in many different tissues. Obesity is characterized by dysfunctional fat deposition leading to a tissue-specific and systemic low-grade inflammation. Androgens and estrogens contribute to the whole adipose tissue inflammatory state, but the involvement of sex steroids in the purinergic signaling modulation in adipocytes is still unknown.

**Methods:** We performed an *in vitro* study to evaluate the possible role of sex hormones on the P2X7R gene expression in human adipocytes, at baseline and after stimulation with bacterial lipopolysaccharide (LPS). We evaluated P2X7R gene expression during *in vitro* differentiation of human adipocytes, in the absence and presence of testosterone (T) and 17β-estradiol (E2) in the presence and absence of LPS. Furthermore, we analyzed the effects of incubation with dihydrotestosterone (DHT), a non-aromatizable androgen, using the co-incubation of isolated human adipocytes with T alone or in combination with anastrozole, an inhibitor of aromatase, the enzyme responsible of T conversion to E2.

**Results:** At baseline, incubation of adipocytes with T or E2 did not significantly affect P2X7R gene expression. On the contrary, the incubation with DHT was associated with a significant reduction of P2X7R gene expression. LPS incubation significantly increased gene expression of P2X7R with respect to baseline. Interestingly, after LPS stimulation, DHT exposure showed an additional effect, markedly increasing the P2X7R gene expression. This amplificatory effect was confirmed by the incubation of adipocytes to both anastrozole and testosterone. In these experimental conditions, while no effect was observed at baseline, an amplification of the expression of the P2X7R mRNA was observed after stimulation with LPS.

**Discussion:** The purinergic system is involved in the inflammatory response of adipocytes, and androgens may modulate its activity. In particular DHT, a non-aromatizable androgen, amplifies the LPS-induced P2X7R gene expression in human adipocytes thus showing a gender regulated response of the expression of this purinergic receptor strongly involved in the inflammatory response in adipose tissue.

## 1 Introduction

Obesity is characterized by abnormal adipose tissue accumulation and ectopic fat deposition due to a condition of energy excess (Ghaben and Scherer, 2019). When adipocytes reach a biological threshold for their expansion they become dysfunctional, leading to the synthesis of hypoxia-induced mediators and pro-inflammatory molecules (Tchernof and Després, 2013). When reaching the circulation, these adipokines promote the development of a chronic, low-grade systemic inflammation responsible for obesity-related comorbidities such as type 2 diabetes and cardiovascular diseases (Bray et al., 2016; Chouchani and Kajimura, 2019). Thus, the identification of the cellular and biochemical pathways involved in adipose tissue dysfunction leading to inflammation could have relevant clinical implications.

The so called “purinergic signaling pathway” is involved in many different biological processes including the immune response (Di Virgilio and Vuerich, 2015). The purinergic pathway encompasses several purines and pyrimidines as signal molecules and different cell surface receptors (Giuliani et al., 2019). Among them, the ATP-sensitive receptor subtype P2X7 (P2X7R) is directly involved in the inflammatory response, being its activation indispensable for the assembly of the NLPR3 inflammasome complex and the release of the potent pro-inflammatory cytokine IL-1β after host exposure to both infectious and non-infectious agents (Giuliani et al., 2017). Consequently, beyond the role in the immune system, considering its ubiquitous cell expression, the P2X7R has been hypothesized to be involved in several human diseases (De Marchi et al., 2016).

Adipocytes have been demonstrated to express different purinergic receptor subtypes (Rossato et al., 2022; Wang and Zhou, 2023), and the role of purinergic signaling in adipose tissue biology has been proposed since the last decade (Burnstock and Gentile, 2018). In particular, P2X7R is considered to primarily contribute to the adipocyte dysfunction in terms of secretion of pro-inflammatory cytokines by these cells after stimulation with extracellular adenosine-tris-phosphate (ATP). ATP is released in a large amount in the extracellular space in certain pathological settings such as hypoxia condition and cell death, both features of the adipose tissue dysfunction; when reaching appropriate concentrations in the extracellular space, ATP can activate P2X7R activating and amplifying the inflammatory cascade (Linden et al., 2019; Vultaggio-Poma et al., 2022). Thus, P2X7R antagonization has been hypothesized as a therapeutic target for the prevention of many different inflammatory disorders including obesity-related complications (Savio et al., 2018; Shokoples et al., 2021). However, it has also to be considered that some physiological conditions may affect the function and the expression of the P2X7R. To this regard, sex hormones are responsible for the different gender-derived susceptibility to both metabolic and immune disturbances. In obesity, sex hormones abnormalities are common, with increasing BMI being responsible of their prevalence (Escobar-Morreale et al., 2017). In the male, obesity is associated with low testosterone plasma levels, which could independently contribute to the inflammatory state as testosterone replacement therapy has shown the potential of reducing circulating inflammatory cytokines plasma levels in hypogonadal subjects (Malkin et al., 2004; Kapoor et al., 2007; Di Vincenzo et al., 2018; Di Vincenzo et al., 2020). However, the effects of the different sex steroids on the purinergic system in adipose tissue have not been investigated yet.

In the present study, we have performed an *in vitro* study investigating the effects of sex hormones in the modulation of the P2X7R expression in human adipocytes.

## 2 Material and methods

### 2.1 Adipocytes isolation and differentiation

Subcutaneous adipose tissue (SAT) was obtained from 5 male subjects, not taking any drugs, undergoing plastic surgery for abdominal wall laxity after weight loss due to bariatric surgery for obesity. The study was conducted according to the guidelines of the Declaration of Helsinki, and approved by the Ethics Committee of the University Hospital of Padova (approval n. RF-2016-02363566). Written informed consent was obtained from each patient.

The stromal vascular fraction was isolated from SAT by collagenase type II digestion (1 mg/mL) at 37°C for 1 h and seeded in DMEM/F12 supplemented with 10% fetal bovine serum (0.35 × 10^6^ cells per well in 24-well plates). The cells were placed in a humidified incubator at a temperature of 37°C and in a 5% v/v CO_2_ atmosphere. After waiting 16-20 h for cell adhesion, a serum-free adipogenic medium containing DMEM/F12 supplemented with 33 μmol/L of biotin, 17 μmol/L of pantothenate, 10 μg/mL transferrin, 66 nmol/L of insulin, 100 nmol/L of dexamethasone, 1 nmol/L of triiodothyronine, 0.25 mmol/L of 3-isobutyl-1-methylxanthine (IBMX), and 10 μmol/L of rosiglitazone was added to the cultures. After 3 days the medium was replaced with adipogenic medium without IBMX and rosiglitazone. The adipogenic medium has been replaced three times a week until the complete differentiation into mature adipocytes.

The differentiation of the vasculo-stromal component in the adipogenic medium was verified by oil-red-O staining, which is used to identify neutral lipids, in particular triglycerides, and by gene expression of Peroxisome Proliferator-Activated Receptor γ (PPARγ), leptin and Fatty Acid Binding Protein-4 (FABP-4), which are traditionally expressed in mature adipocytes (Rossato et al., 2014).

### 2.2 Stimulation of human adipocytes in primary culture

Fully differentiated adipocytes in primary culture as described above were stimulated overnight with 100 nmol/L testosterone (T group), 17 β-estradiol (E2 group), 5α-dihydrotestosterone (DHT group), 200 nmol/L anastrozole (A group), alone or in combination 2 ng/mL LPS 4 h after each sex hormone treatment. All steroids were diluted in dimethyl sulfoxide (DMSO). A control group was incubated with DMSO alone.

### 2.3 RNA extraction and real-time PCR gene expression analysis

RNA was extracted using a specific kit (RNEasy Kit). The amount of RNA recovered by eluting the column with Rnase-free water was evaluated with a spectrophotometer, while the quality of the same was evaluated by a Bioanalyzer. First-strand cDNAs were synthesized from equal amounts of total RNA using random primers and M-MLV reverse transcriptase. The cDNA was used to quantify gene expression levels of the P2X7R gene in the different experimental conditions by using SYBR Green fluorophore. The change in fluorescence at every cycle was monitored and a threshold cycle above background for each reaction was calculated. A melt curve analysis was performed following every run to ensure a single amplified product for every reaction and all reactions were carried out in at least duplicated for every sample. Relative mRNA transcript levels were quantified with the 2^−ΔΔCT^ method using Ribosomal Protein Lateral Stalk Subunit P0 (RPLP0) as housekeeping internal control gene.

### 2.4 Statistical analysis

Statistical analysis was performed using GraphPad Prism software (version 9.5.1, GraphPad Software Inc., San Diego, CA, United States). Results from five separate experiments were considered and expressed as means ± standard deviation (SD). Data comparisons from two samples were analyzed with the Student’s “*t*-test”. Comparisons of means from multiple groups were conducted by ANOVA analysis. The variables were tested for normality using the Shapiro-Wilk test. Differences between groups were considered statistically significant at *p*-value < 0.05.

## 3 Results

### 3.1 DHT reduces the P2X7R gene expression at resting conditions

**FIGURE 1:**
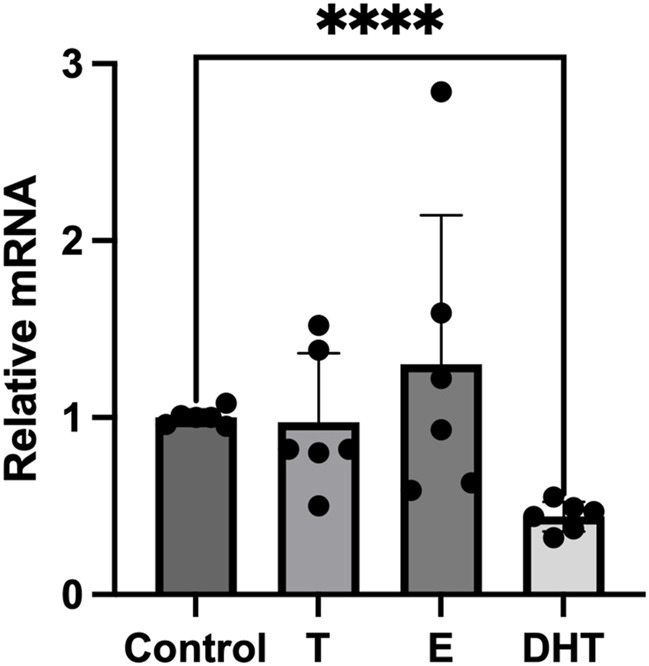
Effects of sex steroids on P2X7 receptor gene expression in human adipocytes. *In vitro* differentiated human adipocytes were incubated with vehicle (Control), testosterone (T, 100 nM), 17β-estradiol (E, 100 nmol/L) and 5α-dihydrotestosterone (DHT, 100 nmol/L). Data are expressed as means ± SD of five independent experiments. ****p < 0.01.

Since sex hormones have been shown to have a modulatory activity on adipocytes inflammatory response, we tested the effects of sex steroid hormones on the expression of P2X7R mRNA in *in vitro* fully differentiated human adipocytes. Adipocytes were stimulated overnight with testosterone (T), 17β-estradiol (E2), and DHT, while control group was incubated with vehicle only. At resting conditions, pre-incubation of adipocytes with T or E2 did not significantly affect the P2X7R gene expression (Figure 1). In particular, T had neutral effect, while E2 slightly increased P2X7R gene expression with respect to control group although without reaching the statistical significance. On the contrary, when adipocytes were exposed to DHT, a non-aromatizable androgen, we observed a significant reduction of the P2X7R mRNA expression with respect to control group.

### 3.2 DHT pre-incubation amplifies the effects of LPS on the P2X7R gene expression

**FIGURE 2:**
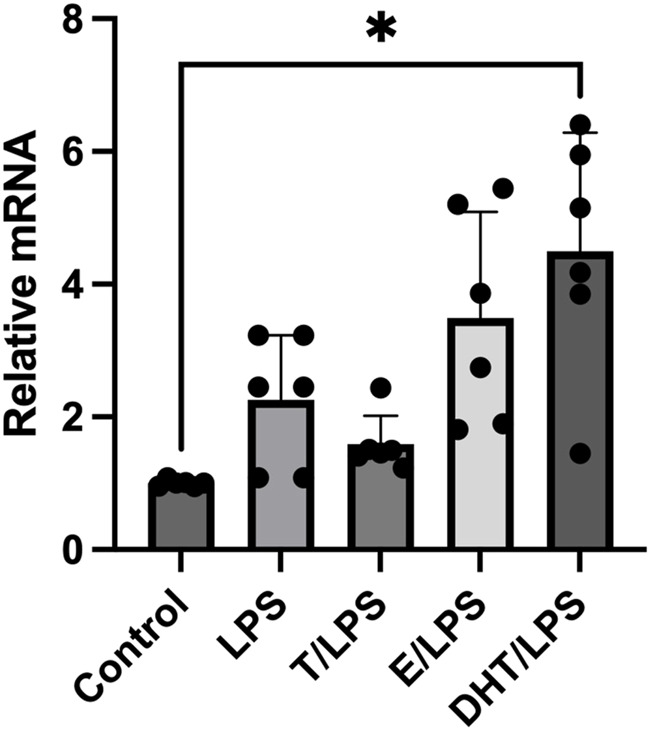
Effects of sex steroids exposure on LPS-stimulated P2X7 receptor gene expression. *In vitro* differentiated human adipocytes were incubated with vehicle (Control), lipopolysaccharide (LPS, 2 ng/mL), 17β-estradiol (E, 100 nmol/L), pre-incubated with testosterone (T, 100 nM) before LPS (2 ng/mL) stimulation (T/LPS), pre-incubated with 17β-estradiol (E, 100 nmol/L) before LPS (2 ng/mL) stimulation (E/LPS), and pre-incubated with 5α-dihydrotestosterone (DHT, 100 nmol/L) before LPS (2 ng/mL) stimulation (DHT/LPS). Data are expressed as means ± SD of five independent experiments. *p < 0.005. Other differences were not statistically significant: LPS vs. LPS/T: *p* = 0.7; LPS vs. LPS/E: *p* = 0.6; LPS vs. LPS/DHT: *p* = 0.2.

To test if human adipocytes response to an inflammatory stimulus were able to increase the P2X7R gene expression, we utilized LPS, a well-known inflammatory stressor also in adipocytes (Jung et al., 2018). As shown in Figure 2, the stimulation of adipocytes with LPS induced a significant increase of the P2X7R gene expression with respect to control (*p* < 0.01). We then evaluated the effects of sex steroids on LPS-stimulated P2X7R gene expression in adipocytes. The pre-incubation of adipocytes with T induced a reduction of the P2X7R gene expression after LPS stimulation, although without reaching the statistical significance. On the contrary, human adipocytes pre-incubation with E2 showed an increased expression of the P2X7R gene after LPS stimulation, significantly higher than that observed stimulating adipocytes with LPS alone (Figure 2). At variance with the observations at resting conditions, adipocytes pre-incubation with DHT induced a significant amplificatory effect on the expression of the P2X7R gene after stimulation with LPS (Figure 2).

### 3.3 Aromatase inhibition preserves the effects of testosterone

**FIGURE 3:**
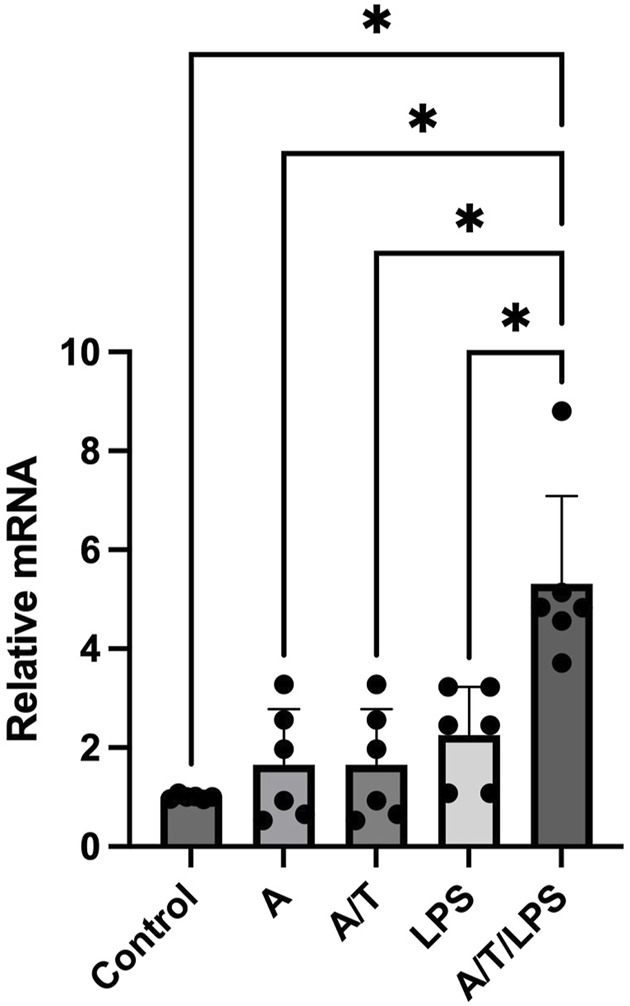
Effects of pre-incubation of human adipocytes with anastrazole/testosterone on LPS-stimulated P2X7 receptor gene expression. *In vitro* differentiated human adipocytes were incubated with vehicle (Control), anastrozole (A, 200 nM), lipopolysaccharide (LPS, 2 ng/mL), pre-incubated with anastrozole (A, 200 nM) before testosterone (T, 100 nM) stimulation (A/T), and pre-incubated with anastrozole (200 nM) and testosterone (T 100 nM) before LPS (2 ng/mL) stimulation (A/T/LPS). Data are expressed as means ± SD of five independent experiments. *p < 0.005.

Incubation of human adipocytes with the aromatase inhibitor anastrozole, did not induce any significant effect on the P2X7R gene expression. Similarly, when human adipocytes were exposed to T after a pre-incubation with anastrozole, there was no significant modification of the P2X7R gene expression. However, when adipocytes were pre-incubated in the presence of anastrozole and T, LPS stimulation induced a significant increase of the P2X7R gene expression in human adipocytes (Figure 3). This increase was greater than that observed when adipocytes were exposed to DHT/LPS as shown in Figure 2.

## 4 Discussion

Purinergic system has been previously hypothesized to play a role in the modulation of adipocyte functions (Burnstock and Gentile, 2018; Rossato et al., 2022). Among the different P2 receptors expressed in adipocytes including human, the P2X7R has been identified as a pharmacological target for the prevention of adipocyte dysfunction in obesity and its related complications (Madec et al., 2011; Rossi et al., 2014; Novak and Solini, 2018; Rossato et al., 2020; Rossato et al., 2022). However, the precise mechanisms regulating the P2X7R expression, activation, and function in the different clinical settings related to the obesity status (fatty liver disease, obstructive sleep apnea, hypogonadism, etc.) remain to be clarified yet. To this respect, it is known that immune response is different in male and female and this sex dichotomy is present also in obesity in many different areas including adipocyte function and metabolic inflammation leading to critical differences in adipose tissue biology. The sex and gender difference in adipose tissue is a factor that should be considered when studying an individuals’ risk for obesity and metabolic dysfunction. This understanding is important for strategizing treatment and prevention measures (Chang et al., 2018; Shepherd et al., 2021).

In the present study we aimed to define the modulation of sex steroid hormones of the expression of LPS induced P2X7R gene expression in *in vitro* fully differentiated human adipocytes. At resting conditions, sex steroids minimally affected the P2X7R gene expression, except for DHT that has been shown to downregulate the P2X7R gene expression in human adipocytes. As expected, adipocytes stimulated with LPS, a product of bacterial wall known to induce the activation of the inflammatory cascade also in human adipocytes (Jung et al., 2018), significantly increased the P2X7R gene expression, demonstrating the involvement of this receptor subtype in the response of adipose tissue to pro-inflammatory stimuli. Interestingly, our results showed that sex steroids modulate the effects of LPS since DHT, a non-aromatizable androgen, amplifies the effect of LPS on the P2X7R gene expression in human adipocytes in culture. These observations were confirmed by the results obtained in human adipocytes pre-incubated with anastrozole, an inhibitor of aromatase, the enzyme responsible of T transformation in E2.

Sex steroids affect the metabolic and inflammatory responses of adipose tissue (Law et al., 2014; Chang et al., 2018; Shepherd et al., 2021). In hypogonadal men testosterone replacement therapy has been shown to improve markers of glucose metabolism impairment and to reduce serum levels of inflammatory cytokines (Malkin et al., 2004; Kapoor et al., 2007). Generally, the relationship between obesity and hypogonadism in the male has been considered due to the aromatization of circulating testosterone occurring in the enlarged adipose depots expressing huge amounts of aromatase, resulting in abnormal androgen/estrogen ratio (Di Vincenzo et al., 2018). Thus, testosterone supplementation and aromatase inhibition are expected to be somehow protective with respect to the metabolic homeostasis and inflammatory response by adipose tissue. In this sense, our results showing an increased LPS-induced P2X7R gene expression after DHT and A/T exposure are in contrast with what expected. However, recent studies questioned the role of androgens aromatization in the development of adipose tissue dysfunction, shedding new light in this research area. In particular, Ohlsson et al. have recently demonstrated in male experimental animals that the overexpression of aromatase induced the reduction of adipose tissue inflammation and an improvement of insulin sensitivity (Ohlsson et al., 2017). These Authors explained those results by hypothesized peculiar effects of estrogens on adipocytes, suggesting that a normal enzymatic activity is necessary for a normal adipose tissue biology (Ohlsson et al., 2017). Furthermore, it is known that aromatase gene inactivating mutations in men are associated with glucose abnormalities which are reverted by estrogen supplementation (Maffei et al., 2004), suggesting that a normal E/T ratio and a functional sex steroid feedback loop regulation is necessary to maintain metabolic homeostasis and, as a consequence, even a normal inflammatory reaction.

Furthermore, no reports are available regarding the relationships between sex steroids and the purinergic system in obesity. Human adipocytes have been demonstrated to express functional active P2X7R, with an upregulated expression in subjects with metabolic syndrome which may contribute to the associated chronic inflammatory state (Madec et al., 2011; Rossato et al., 2022). However, in animal models, P2X7R seems to be involved also in the sex-dependent adipose tissue distribution and adipocytes differentiation. Infact, the P2X7R knockout male mice shows weight gain, abnormal fat distribution and ectopic lipid accumulation (Beaucage et al., 2014). These results are supported by the recent studies showing a reduction of energy expenditure in P2X7R-KO mice (Giacovazzo et al., 2018; Sarti et al., 2021). Thus, it is possible that there is a sex dichotomy in the P2X7R functions in human adipocytes regarding their inflammatory and metabolic (meta-inflammatory) activity. Moreover, sex steroids may drive different effects in normal and abnormal (es. inflammatory status) conditions, as represented in our experiments showing that in human adipocytes a non-hydrolizable androgen such as DHT reduces the expression of the P2X7R gene at resting conditions, while increasing P2X7R gne expression after LPS exposure.

The translation of these concepts in the clinical practice could lead to hypothesize that in the obese male, testosterone replacement therapy might prevent the adipocyte dysfunction modulating the P2X7R gene expression in these cells. On the contrary, the detrimental effects of androgen supplementation might arise in the presence of conditions characterized by an increased activation of the inflammatory cascade.

At the best of our knowledge, this is the first report on the modulation of P2X7R gene expression by non-aromatizable androgen in human adipocytes. The present study has also some limitations: first of all, this is an *in vitro* study, and these results might be not directly transferred *in vivo*, despite the use of human adipocytes. Second, we obtained adipose tissue samples from subcutaneous adipose tissue, while *in vivo* the visceral adipose tissue is more biologically active in the induction of the detrimental complications of obesity (Alexopoulos et al., 2014; Kawai et al., 2021). However, in our opinion this work further suggests the complex role of adipose tissue in the inflammatory response of the body, supporting the need of additional *in vitro* and above all *in vivo* studies above all considering the possible existence of a gender dichotomy in the inflammatory response within the adipose tissue in obesity.

## 5 Conclusion

The inflammatory response generally shows a sexual dimorphism and the sex hormones have been pointed as the main responsible for these gender-related differences. In the present study we show that the P2 purinergic-regulated inflammatory response of adipose tissue could be modulated by sex steroids. In particular, we demonstrated that exposure to non-aromatizable testosterone influences the expression of the P2X7R gene in human-derived adipocytes in a bi-modal manner, with a reduced expression at basal condition, and an increased expression after stimulation with a potent inflammatory stimulus such as LPS. These observations suggest to carefully consider the specific androgen replacement therapy in hypogonadal male patients and suggest that the P2X7R might represent a relevant therapeutic target to treat the obesity-related inflammatory state and its related complications.

## Data Availability

The raw data supporting the conclusion of this article will be made available by the authors, without undue reservation.
